# Selections

**Published:** 1891-11

**Authors:** 


### Selections.

### LOCAL TREATMENT OF DYSENTERY

There seems to be in modern medical thought a very strong tendency to consider disease as constitutional rather than local. I do not doubt but that there are one or more forms of dysentery dependent upon the presence of poisons in the blood, but I feel very confident that the dysentery, as we see it ordinarily in this climate, is essentially a local inflammation, independent of any blood poisoning. If this be true, the disease should be especially amenable to local treatment. It is true that the ordinary treatment, which seems not to be local, really owes much of its efficiency to a local influence. Thus, the purgative acts by a purely local depletion; the mercurial, or the ipecac, by a local stimulation of the glands involved; whilst the bismuth spreads itself upon the mucous membranes and by its local action lessens inflammation. It has seemed to me, however, worth while to draw the attention of practitioners to the value of the direct application of remedial agents to the affected parts.

Many years ago I published a series of cases of chronic dysentery, demonstrating the extraordinary efficiency of forced enemata containing one-half a drachm to a drachm of nitrate of silver dissolved in two or three quarts of water, and further experience has corroborated all that I said. Indeed, from time to time have appeared papers in the medical journals proposing the treatment as both novel and efficacious.

In acute dysentery, involving the colon high up, I have found large enemata, containing two or three drachms of subnitrate of bismuth, much more efficient than the exhibition of bismuth by the mouth. When the symptoms are severe, this local treatment may often be preceded with advantage by washing out the colon with large quantities of cold water. I have never used injections of nitrate of silver in acute dysentery, although the effect of the local application of the nitrate in other inflammations of mucous membranes would justify trial of the remedy. I have seen, in one or two cases, large enemata of very hot water injected without affording relief, and believe that hot water enemata are, in their ordinary results, not at all comparable with large injections of ice-cold water.

When the lower part of the colon is affected, the local use of ice sometimes has an almost marvelous effect. I have, indeed, seen the whole aspect of a very severe and alarming case, in which the symptoms indicated that the colon was affected high up, changed in a single hour by the continuous use of *ice suppositories.* While it is not necessary to have the pieces of ice entirely regular in shape, care should be exercised that no sharp edges are left. The suppositories should be rapidly used, one being put into the rectum every three to five minutes, so as to get, for at least half an hour to an hour, the effect of the continuous application of cold.

When the tenesmus is very severe, iodoform suppositories are often much more efficient than opium in bringing relief.

A remedy which has been from time to time recommended very highly in dysentery, but has not, I think, been much used, is ergot; and when the passages contain large quantities of blood, or are nearly pure blood, the extract of ergot would seem to be indicated. I have never myself used ergot by the mouth in these cases, but have employed suppositories containing twelve grains of extract of ergot and four grains of iodoform, used every two hours until four or five suppositories had been taken with, seemingly, great advantage.

I do not mean to advocate the local treatment of dysentery as a substitute for the use of mercurials, purgatives and ipecacuanha, etc., but as a very important adjuvant to the older forms of treatment. Nevertheless, in my experience, the effect of local remedies has been more prompt and decided than that of drugs given by the mouth; but in cases of any severity the attack upon the disease may be made from each end of the mucous tract.

As illustrating this method of treatment, I give brief outline of a case seen very recently.

This was a middle-aged woman, an habitual patient of my own, who had been suddenly seized with violent abdominal pain and diarrhœa four days previous, during my temporary absence from the city, and came under the care of another practitioner of repute, by whom she was treated with mercurials, etc., *secundum artem.* I was sent for about 6 A. M., and found her in a condition of marked prostration, suffering from great tormina and tenesmus. She had had during the night six passages which were said to have been of the character of the one shown me. This consisted of about a teacupful of blood, mixed with just enough sero-mucous liquid to prevent clotting. Calomel was ordered by the mouth in one-sixth grain doses. Ice suppositories were freely used for half an hour, with great immediate relief to pain, and were followed at once by suppositories containing five grains of iodoform and twelve grains of extract of ergot, given every hour until four were taken. Two hours after the commencement of the local treatment the passages were free from blood. By afternoon the patient was without any abdominal distress, and the bowels were quiet; but during the night the calomel produced several characteristic fæcal discharges, containing a few scybala, on account of which a dose of castor oil was given. The operation of the oil ended the case so far as abdominal symptoms were concerned, and in sixty hours after the commencement of the treatment the patient had a normally-formed fæcal passage.—*Dr. H. C. Wood, Uni. Med. Magazine.*

---

GONOCOCCI. Drs. Vibert and Bordas. (*La Med. Moderne*, Nov. 13th, 1890; Jan. 1st, 1891; and *Journ. des Mal. Cut.*, June, 1891.)

In the first article the authors attempt to show that the gonococcus has no value as a diagnostic sign in medico-legal cases where the nature of a vulvitis is to be determined. They found, to all appearances, indentical organisms in six instances where blennorrhagia in young girls was attributable to other than venereal cause. In the second paper are reported the successful attempts of the authors to cultivate the gonococcus. Positive results were obtained upon bouillon, agar, and potato. The cultivations showed diplococci in all respects similar to the gonococci. Thus it would appear that cultivation is not sufficient to make the diagnosis absolute. If the observations of different authors are compared, it will be seen that they are not in accord either as to the best media for cultivations, the proper temperature, the length of time required, or the appearance of the colonies of cocci after they have developed. In the author's own hands the results have not been uniform.

They are, hence, of opinion that in the present state of knowledge it is impossible to recognize the gonococcus with absolute certainty, and to distinguish it from other micrococci to be found in vaginal secretions. In the last article, upon "the gonococcus in legal medicine," it is stated that in the authors' experience only one variety of micrococcus is found in the acute vulvitis of little girls; and this presents all the features of the gonococcus.

In conclusion, they feel justified in the statement that at the present time the question of the gonococcus is far from being solved with that complete certainty which forensic medicine requires, and believes that in no case is the expert authorized in affirming the blennorrhagic nature of a vulvitis based upon a bacteriological examination, no matter how complete.—*Journal of Cut. and Ven. Diseases.*

---

PRESENT STATUS OF BRAIN SURGERY.—At the recent American Surgical Association, in Washington, Dr. D. Hayes Agnew, of Philadelphia, presented a paper with this title. Following are his conclusions:

1. That all fractures of the skull attended with depression, however slight, and entirely irrespective of symptoms, should, in view of the late after-affects, be subjected to the trephine. 2. That trephining for traumatic epilepsy promises only palliation at best. 3. That trephining for Jacksonian epilepsy is to be regarded as only affording temporary benefit. 4. That trephining for abscess, in view of the fact that all such cases left alone almost invariably terminate fatally, is entirely proper, and that the earlier such operation is done the better. 5. That trephining for intra-cranial traumatic hemorrhage is both an imperative and highly promising operation. 6. That trephining for cephalalgia, or traumatic epilepsy (medical measures having failed), should be undertaken with every prospect of success. 7. That trephining for hydrocephalus is a useless operation. 8. That trephining for microcephalus, independent of athetosis, confers no credit upon surgery. 9. That it is more than probable that as our observations multiply the sphere of the trephine as a preliminary for the removal of brain tumors will be lessened rather than be amplified.

---

AN EXTREME CASE.—At the meeting of the Obstetrical Society, Mr. M. Shield read a paper upon a case of extra-uterine gestation associated with sloughing of the abdominal wall and attempted extrusion of a matured and putrid fœtus near the umbilicus. The patient, a young married woman, a primipara, had been ill with fever, and for some months there had been a large abdominal tumor. Upon exploration, the uterus was found empty. Near the umbilicus there was a considerable ovoid opening with sloughing margins. Through this protruded a tumor somewhat larger than a cricket-ball; at its somewhat constricted base it was firmly grasped by the surrounding tissues; the tumor was black and offensive. The patient was placed under chloroform, and the opening enlarged downward, and a fœtus was extracted along with much fœtid fluid and gas, followed by bright blood. The placenta was deeply attached above and behind, and the sac appeared to be extra-peritoneal. The hæmorrhage was stopped by hot water irrigation. The placenta was removed piecemeal, further bleeding being prevented by packing with sponges. The patient made a good recovery.—*American Lancet, London Letter.*

---

DIAGNOSIS OF TUBERCULAR MENINGITIS IN CHILDREN.—At a recent meeting of the American Pædiatric Association, Dr. W. P. Northrup read an interesting paper on this subject. He gave four symptoms which, when they existed together, were to him convincing evidence of the disease—persistent vomiting, irregular pulse, irregular breathing and apathy; there were also other significant symptoms connected with the organs of special sense. Professor Jacobi agreed with Dr. Northrup in the importance of the persistent vomiting as a diagnostic sign; the vomiting is apt to be marked when the meninges of the base of the brain are the seat of the tubercular deposit; if the tubercular deposit is not marked in this region the vomiting is apt to be less pronounced or absent. Distinction must be made between the cerebral type of vomiting, which is projectile and not accompanied by nausea, and that which is merely reflex or of gastric origin. Dr. Northrup traced the infection in one of his reported cases to the use of tuberculous milk.—*American Journal Medical Sciences.*

---

SCIATICA.—Concerning this disease, Dr. Eliot, New Haven, Connecticut, makes the following suggestions (*New York Medical Journal*):

1. A large proportion of cases of sciatica are neuritis, and not simply neuralgia.

2. Temporary relief of suffering should be secured by hypodermic injections of morphine and atropine, or of theine.

3. Among curative agents, salicylate of sodium and icdide of potassium are especially valuable—the former in acute, the latter in chronic cases.

4. Considerable benefit may often be derived from the administration of the more purely neurotic drugs—aconite, belladonna, and gelsemium, as:

R. Ext. belladon., fl. ℨss.

Ext. aconiti., fl. ℨiss.

Ext. gelsem., fl. ad ℥vi.

M. Sig.—Give six, seven or eight drops every four hours.

5. Cantharidal blisters are of great service in promoting the cure of the disease, when used in conjunction with appropriate internal treatment.

ACUTE BRONCHITIS.—Dr. H. C. Wood:

℞. Potass. Nitrat........................ 1 ounce.

Suc. limonis........................ 2 fl. ounces.

Syr. ipeac........................ ½ fl. ounce.

Syr.............................. 6 fl. ounces.

M. Sig.—A tablespoonful four to six times a day.

---

HYDRASTIS CANADENSIS IN NIGHT SWEATS.—Dr. Cruse relates in the *All. Med. Zeitung* an observation made on the above drug. He employed hydrastis for a case of hæmoptysis and observed that night sweats did not come on as usual. Patient was in the last stage of phthisis. This led to his trying hydrastis for the night sweats themselves. He gave thirty minims of the fluid extract, and always with complete success. And what is more, the sweats kept off when the hydrastis had been omitted for three weeks. These results were confirmed by trials in a number of cases.—*Lancet Clinic.*

---

ECZEMA OF THE FACE AND SCALP.—Crusts should first be softened with olive oil, and after they are removed the surface should be anionted with the following ointments :

℞. Ac. boric, 45 grains.

Zinc oxide, 75 grains.

Vaseline, Starch, } of each 450 grains.

Eliminate fatty materials from the diet, and combat habitual constipation with appropriate enemata.—*Medical News.*

---

A DEATH under ether occurred in New York, September 9th. The patient was a man, fifty years of age, who was to be operated upon for removal of a tumor of the side. He was examined previous to the administration of the ether, and presented no symptoms of heart trouble.—*Med. Record.*

The Southern Surgical and Gynecological Association will meet in Richmond, Va., Nov. 10th, 11th and 12th.

Following is a partial list of papers to be read: The President's Annual Address, Louis S. McMurtry, M. D., Louisville, Ky.; Remarks on Systemic Infection from Gonorrhœa, Illustrated by Cases, Bedford Brown, M. D., Alexandria, Va.; The Rational Treatment of Peritonitis Based upon the Consideration of the Pathological Conditions Present, W. D. Haggard, M. D., Nashville, Tenn.; A Medico-Legal Aspect to Pelvic Inflammation, W. W. Potter, M. D., Buffalo, N. Y.; Complications in Pelvic Surgery, and How to Deal with Them, Joseph Price, M. D., Philadelphia, Pa.; Cholecystotomy—Report of Case—52 Gallstones and 10 Ounces of Pus Removed—Success, W. B. Rogers, M. D., Memphis, Tenn.; Some of the Complications of Psoas Abscess, J. McFadden Gaston, M. D., Atlanta, Ga.; Laparotomies Performed in the Past Year, Thomas Opie, M. D., Baltimore, Md.; Imperforation of the Rectum, Geo. Ben. Johnston, M. D., Richmond, Va.; A Case of Induced Abortion for the Relief of the Nausea, and Vomiting of Pregnancy, with Remarks, Christopher Tompkins, M. D., Richmond, Va.; The Principle of Drainage as Applied to Surgery of the Deep Urethra, F. W. McRae, M. D., Atlanta, Ga.; The Neuroses of the the Genito-Urinary System in the Male, Frank Lydston, M. D. Chicago, Ill.; Nephrectomy, with Report of Cases, Edwin Ricketts, M. D., Cincinnati, O.; Venomous Serpents of the United States, and the Treatment of Wounds Inflicted by Them, Paul B. Barringer, M. D., University of Virginia.; A Report of Some Additional Cases of External Perineal Urethrotomy without a Guide, J. Edwin Michael, M. D., Baltimore, Md.; Growth of Fibroid Tumors of the Uterus, after the Menopause, Jos. Taber Johnson, M. D., Washington, D. C.; The Part the Shoulders Play in the Production of Laceration of the Perineum, with Suggestions for its Prevention, W. D. Haggard, M. D., Nashville, Tenn.; The Pedicle in Hysterectomy; How Formed; Its Subsequent Behavior; Its Final Condition, I. S. Stone, Washington, D. C.; A Case of Pelvic Abscess, John Brownrigg, M. D., Columbus, Miss.; A Case of Cyst of the Mesentery, with Remarks, J. A. Goggans,

M. D., Alexander City, Ala.; The Female Urethra, K. P. Moore, M. D., Macon, Ga.; Medico-Legal Aspect of Intestinal Surgery, J. D. S. Davis, M. D., Birmingham, Ala.; Albuminuria; Its Relation to Surgical Operations, J. W. Long, M. D., Randleman, N. C.; Senile Gangrene, Frank Prince, M. D., Bessemer, Ala.; Hemorrhage *versus* Shock, W. L. Robinson, M. D., Danville, Va.; Treatment of Gallstone, with Report of Cases, W. E. B. Davis, M. D., Birmingham, Ala.; (Title of paper not determined), Hunter McGuire, M. D., Richmond, Va.; (Title of paper not determined), Duncan Eve, M. D., Nashville, Tenn.; (Title of paper not determined), A. V. L. Brokaw, M. D., St. Louis, Mo.; (Title of paper not determined), Chas. A. L. Reed, M. D., Cincinnati, O.; (Title of paper not determined), W. F. Westmoreland, M. D., Atlanta, Ga.

LOUIS S. MCMURTRY, M. D., President.

W. E. B. DAVIS, M. D., Secretary.

---

MEMBERSHIP IN THE AMERICAN MEDICAL ASSOCIATION.— This is obtainable, at any time, by a member of any State or local Medical Society which is entitled to send delegates to the Association. All that is necessary is for the applicant to write to the Treasurer of the Association, Dr. Richard J. Dunglison, Lock Box 1274, Philadelphia, Pa., sending him a certificate or statement that he is in good standing in his own Society, signed by the President and Secretary of said Society, with five dollars for annual dues. Attendance as a delegate at an annual meeting of the Association is not necessary in order to obtain membership. On receipt of the above amount the weekly *Journal* of the Association will be forwarded regularly.

---

DR. CHRISTOPHER JOHNSTON, one of the most prominent surgeons of Baltimore, died October 11th, in the sixty-ninth year of his age. He was, at the time of his death, Professor of Surgery in the University of Maryland.

